# Assessment of anti-PD-(L)1 for patients with coexisting malignant tumor and tuberculosis classified by active, latent, and obsolete stage

**DOI:** 10.1186/s12916-021-02194-z

**Published:** 2021-12-20

**Authors:** Shan Su, Mei-Feng Ye, Xiao-Ting Cai, Xue Bai, Zhi-Hao Huang, Si-Cong Ma, Jian-Jun Zou, Yu-Xiang Wen, Li-Juan Wu, Xue-Jun Guo, Xian-Lan Zhang, Wen-Chang Cen, Duo-Hua Su, Hui-Yi Huang, Zhong-Yi Dong

**Affiliations:** 1 Department of Oncology, Guangzhou Chest Hospital, Guangzhou, China; 2 Department of Radiation Oncology, Nanfang Hospital, Southern Medical University, Guangzhou, China; 3 Department of Tuberculosis, Guangzhou Chest Hospital, Guangzhou, China

**Keywords:** Malignant tumor, Tuberculosis, PD1/PD-L1, Efficacy, Safety

## Abstract

**Background:**

It is not a rare clinical scenario to have patients presenting with coexisting malignant tumor and tuberculosis. Whether it is feasible to conduct programmed death-(ligand) 1 [PD-(L)1] inhibitors to these patients, especially those with active tuberculosis treated with concurrent anti-tuberculosis, is still unknown.

**Methods:**

This study enrolled patients with coexisting malignancy and tuberculosis and treated with anti-PD-(L)1 from Jan 2018 to July 2021 in 2 institutions. The progression-free survival (PFS), objective response rate (ORR), and safety of anti-PD-(L)1 therapy, as well as response to anti-tuberculosis treatment, were evaluated.

**Results:**

A total of 98 patients were screened from this cohort study, with 45 (45.9%), 21 (21.4%), and 32 (32.7%) patients diagnosed with active, latent, and obsolete tuberculosis, respectively. The overall ORR was 36.0% for anti-PD-(L)1 therapy, with 34.2%, 35.5%, and 41.2% for each subgroup. Median PFS was 8.0 vs 6.0 vs 6.0 months (*P*=0.685) for each subgroup at the time of this analysis. For patients with active tuberculosis treated with concurrent anti-tuberculosis, median duration of anti-tuberculosis therapy was 10.0 (95% CI, 8.01–11.99) months. There were 83.3% (20/24) and 93.3% (42/45) patients showing sputum conversion and radiographic response, respectively, after anti-tuberculosis therapy, and two patients experienced tuberculosis relapse. Notably, none of the patients in latent and only one patient in obsolete subgroups showed tuberculosis induction or relapse after anti-PD-(L)1 therapy. Treatment-related adverse events (TRAEs) occurred in 33 patients (73.3%) when treated with concurrent anti-PD-(L)1 and anti-tuberculosis. Grade 3 or higher TRAEs were hematotoxicity (*n* = 5, 11.1%), and one patient suffered grade 3 pneumonitis leading to the discontinuation of immunotherapy.

**Conclusions:**

This study demonstrated that patients with coexisting malignant tumor and tuberculosis benefited equally from anti-PD-(L)1 therapy, and anti-tuberculosis response was unimpaired for those with active tuberculosis. Notably, the combination of anti-PD-(L)1 and anti-tuberculosis therapy was well-tolerated without significant unexpected toxic effects.

**Supplementary Information:**

The online version contains supplementary material available at 10.1186/s12916-021-02194-z.

## Background

Tuberculosis and cancer represent two major challenges in health care worldwide, especially in China, where the burden of tuberculosis remains the third highest globally, and the coexistence of tuberculosis and cancer is not uncommon [1–3]. The relationship between tuberculosis and cancer is complex [4]. Multiple studies have demonstrated that cancer is a risk factor for tuberculosis activation and tuberculosis itself also promotes cancer, especially lung cancer [4–9].

The safety and efficacy of anti-cancer therapy in patients with coexisting malignancy and tuberculosis treated with concurrent anti-tuberculosis had been investigated in previous studies [10–12]. Recently, we also confirmed the clinical benefits of the concurrent anti-tuberculosis and chemotherapy in lung cancer patients with co-existent tuberculosis [13]. Compared to conventional anti-cancer regimens, immune checkpoint inhibitors (ICIs) have proven to offer superior survival benefits and fewer adverse events [14], thus making them an emerging therapeutic option for cancer patients and even first-line treatment for patients with some advanced-stage malignancies. However, patients with autoimmune disease or chronic infectious disease including tuberculosis were routinely excluded from trials of checkpoint immunotherapies, as ICIs may cause host immune imbalance and develop immune-related adverse events (irAEs) [15].

A recent study had shown that ICIs could be a treatment option for patients with lung cancer and a history of tuberculosis [16]. However, whether ICIs could be used for patients with coexisting malignancy and tuberculosis, especially for those with active tuberculosis treated with concurrent anti-tuberculosis, remains unknown. Moreover, programmed cell death 1 (PD-1) and programmed cell death ligand 1 (PD-L1) pathways had been proven to be crucial in controlling excessive inflammation in tuberculosis, while deficiency in PD-1 would lead to deterioration of tuberculosis in animal models [17–19]. Therefore, whether anti-PD-(L)1 would induce treatment failure in anti-tuberculosis regimen or even result in tuberculosis re-activation remains to be an unresolved concern for clinicians.

In this study, we assessed the efficacy and safety of anti-PD-(L)1 treatment in patients with coexisting malignancy and tuberculosis and investigated the efficacy of anti-tuberculosis therapy in those with active tuberculosis.

## Methods

### Patients

**Fig. 1:**
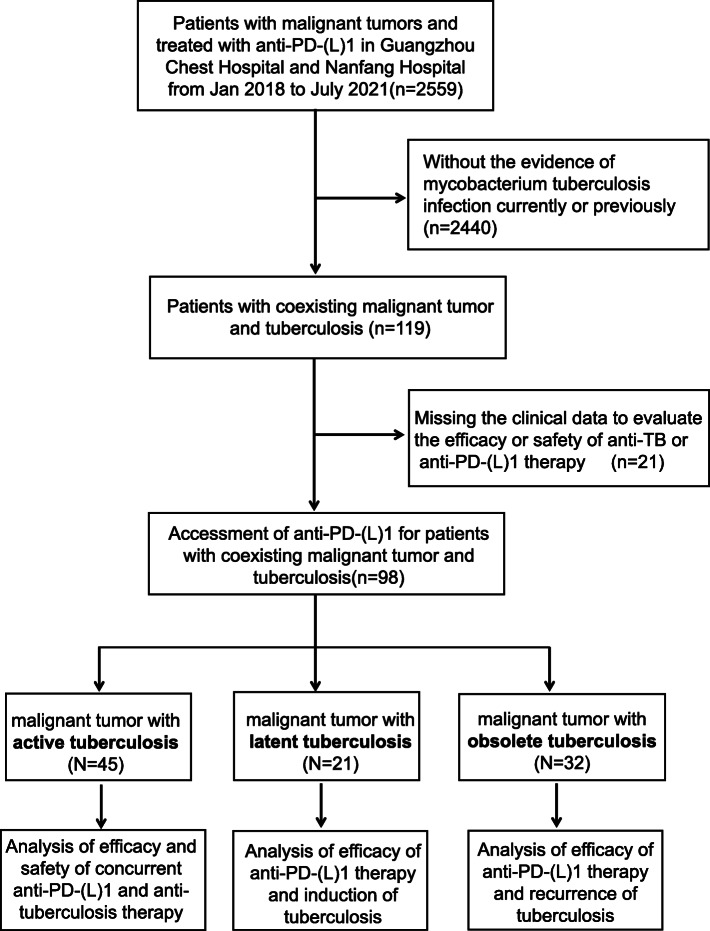
CONSORT flow diagram.

Patients diagnosed with coexisting malignant tumor and tuberculosis and treated with anti-PD-(L)1 were enrolled in Guangzhou Chest Hospital and Nanfang Hospital from Jan 2018 to July 2021. Based on the status of tuberculosis, patients were divided into three groups: active tuberculosis, latent tuberculosis, and obsolete tuberculosis. Diagnosis of active tuberculosis was confirmed by bacteriologic, pathologic, radiographic, or clinical evidence. Latent tuberculosis infection (LTBI) was defined as positive interferon gamma-released assay (IGRA) without evidence of clinically manifest active tuberculosis. The definition of obsolete tuberculosis was as follows: no clinical symptom or signs related to active tuberculosis, negative bacteriological examination, and radiographic calcified, strip-shaped, or sclerosis lesions [20]. Patients with suspected tuberculosis and nontuberculous mycobacteria were excluded. Patients who completed anti-TB treatment but still harbored residual potential active lesions on their CT scanning were also included in the active tuberculosis group [21]. Patients missing efficacy or safety evaluation of anti-tuberculosis or anti-PD-(L)1 therapy were also excluded (Fig. 1).

### Study design

This was a double-center, retrospective analysis of patients with coexisting malignancy and tuberculosis who were treated with anti-PD-(L)1. Baseline patient characteristics were collected, including age, sex, smoking status, pathological types, tumor stage, PD-L1 status, anti-PD-(L)1 treatment information, diagnosis, and classification of tuberculosis. The treatment efficacy of anti-PD-(L)1 therapy was defined by the Response Evaluation Criteria in Solid Tumors (RECIST)1.1 [22], which included objective response rate (ORR), duration of response (DOR), disease control rate (DCR), and progression-free survival (PFS). In terms of defining treatment efficacy of anti-tuberculosis therapy, we assessed the sputum conversion, radiographic response, and tuberculosis relapse condition. The definition of radiographic tuberculosis absorption was reduction of 1/2 or more of the tuberculosis lesions or reduction in cavity size by 1/2 in radiographic examination [23, 24]. Treatment-related adverse events (TRAEs) were defined as adverse events of any grade that were not present at baseline or had progressed from baseline during anti-PD-(L)1 and anti-tuberculosis therapies, according to the National Cancer Institute Common Terminology Criteria for Adverse Events (CTCAE version 4.03).

This retrospective cohort study was approved by the institutional review board of Guangzhou Chest Hospital and Nanfang Hospital. Patients had been informed of the possible risks and alternative treatment options before the anti-PD-(L)1 therapy or anti-tuberculosis treatment. All the patients had signed the informed consent for treatments.

### Statistical analysis

Categorical and continuous variables were analyzed using descriptive statistics, listed as frequency (percentage) and median (range), respectively. ORR was defined as the sum of complete and partial response. DCR was defined as the sum of ORR and stable disease. DOR was defined as the time from first documented response to the date of progression after the anti-PD-(L)1 therapy. PFS was defined as the time from the first dose of anti-PD-(L)1 therapy to disease progression or death. All patients were censored at last available follow-up. DCR and PFS were estimated using the Kaplan-Meier method and reported with 95% confidence interval (CI). The log-rank test was used to compare PFS distribution among the patients with active tuberculosis, latent tuberculosis, and obsolete tuberculosis. All analyses were performed by GraphPad Prism, version 9.1.0.211. *P* < 0.05 was considered statistically significant.

## Results

### Study population and baseline characteristics

A total of 2559 patients were diagnosed with malignant tumor and treated with anti-PD-(L)1 therapy between January 2018 and July 2021, and 98 patients with coexisting malignant tumor and tuberculosis were included and divided into three groups, active tuberculosis (*n*=45), latent tuberculosis (*n*=21), and obsolete tuberculosis (*n*=32), for final analyses (Fig. 1). Baseline characteristics are shown in Table 1. Patients with malignancy in this cohort were mostly diagnosed with non-small cell lung cancer (NSCLC) with 79.4% in total population and 71.1%, 85.6%, and 87.5% for the above three subgroups, respectively. Most patients received the first-line ICI therapy with combined regimen of anti-PD-(L)1 therapy plus chemotherapy. For the diagnosis of tuberculosis, in the active tuberculosis group, most patients (39/45, 86.7%) were secondary pulmonary tuberculosis (PTB), followed by tuberculous pleuritis (5/45, 11.1%) and hematogenous PTB (1/45, 2.2%) with confirmed bacteriologic (36/45, 80.0%) or pathologic evidence (4/45, 8.9%). All patients in the latent tuberculosis group were diagnosed with immunological evidence with positive IGRA, and all patients in the obsolete tuberculosis group were diagnosed based on radiological evidence with negative bacteriological examination (Table 1).

**Table 1:** Baseline characteristics (*N*=98)

Characteristics	Tumor and active TB, ***n*** (%)	Tumor and latent TB, ***n*** (%)	Tumor and obsolete TB, ***n*** (%)
**Median age (range)**	62 (29–72)	65 (51–81)	66 (22–80)
**Men**	40 (88.9)	20 (95.2)	31 (96.9)
**Smoking**			
Current/former	29 (64.4)	17 (80.9)	26(81.3)
Never	16 (35.6)	4 (19.1)	6 (18.7)
**Cancer types**			
NSCLC	32 (71.1)	18 (85.6)	28(87.5)
SCLC	1 (2.2)	1 (4.8)	4 (12.5)
ESCC	7 (15.6)	1 (4.8)	0
CSCC	1 (2.2)	0	0
HNSCC	1 (2.2)	0	0
HCC	2 (4.5)	0	0
BC	1 (2.2)	0	0
RC	0	1 (4.8)	0
**Stage**			
III	11 (24.4)	9 (42.9)	6 (18.7)
IV	34 (75.6)	12 (57.1)	26 (81.3)
**ICI therapy**			
Adjuvant	2 (4.4)	2 (9.5)	3 (9.4)
1st line	21 (46.7)	15 (71.4)	21 (65.6)
2nd line	16 (35.6)	3 (14.3)	6 (18.7)
3rd line	6 (13.3)	1 (4.8)	2 (6.3)
**Treatment regimen**			
Anti-PD-(L)1 monotherapy	12 (26.7)	2 (9.5)	8 (25.0)
Anti-PD-(L)1 + chemotherapy	33 (73.3)	19 (90.5)	24 (75.0)
**PD-L1 status**			
Positive	8 (17.8)	5 (23.8)	13 (40.6)
Negative	4 (8.9)	2 (9.5)	4 (12.5)
Unknown	33 (73.3)	14 (66.7)	15 (46.9)
**Diagnosis of TB**			
Clinical	5 (11.1)	0	0
Bacteriologic	36 (80)	0	0
Pathologic	4 (8.9)	0	0
Immunological	0	21 (100)	0
Radiological	0	0	32 (100)

Abbreviation: *NSCLC* non-small cell lung cancer, *SCLC* small cell lung cancer, *ESCC* esophageal squamous cell carcinoma, *CSCC* cutaneous squamous cell carcinoma, *HNSCC* head and neck squamous cell carcinoma, *HCC* hepatocellular carcinoma, *BC* breast cancer, *RC* rectal cancer, *ICI* immune checkpoint inhibitors

### Efficacy of anti-PD-(L)1 treatment

**Fig. 2:**
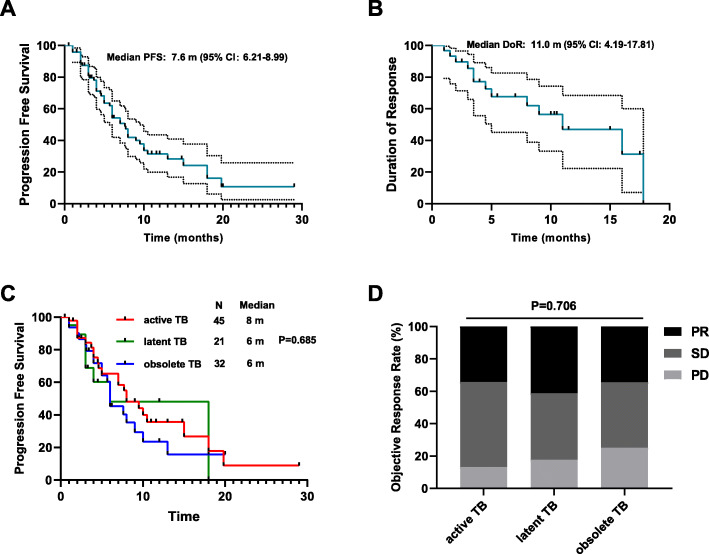
Efficacy of anti-PD-(L)1 therapy. Kaplan-Meier curves for **A** progression-free survival and **B** duration of response among the total patients who received anti-PD(L)1 therapy. **C** Progression-free survival was measured to compare active tuberculosis patients who received anti-tuberculosis therapy with patients diagnosed with latent or obsolete tuberculosis who received no anti-tuberculosis therapy. **D** Proportional representation of objective response rate in the above three groups

The median PFS was 7.6 months [95% confidence interval (CI), 6.21–8.99]. We further explored whether the history of tuberculosis would affect the duration of response in patients achieving partial response or complete response. The median DOR was 11.0 months (95%CI, 4.19–17.81) for the total population with a median follow-up of 11.0 months (95%CI, 8.13–13.87) (Fig. 2A, B). To further clarify whether the efficacy of anti-PD-(L)1 therapy was affected by the tuberculosis activity status or anti-tuberculosis therapy, we compared the oncologic outcomes of active tuberculosis patients who received anti-tuberculosis therapy with those diagnosed with latent and obsolete tuberculosis who did not receive anti-tuberculosis therapy. Notably, there was no statistical significance among the three groups in terms of both PFS (8.0 vs 6.0 vs 6.0 months, *P* = 0.685; Fig. 2C) and ORR (34.2%, 41.2% and 34.4%, *P* = 0.706; Fig. 2D).

**Fig. 3:**
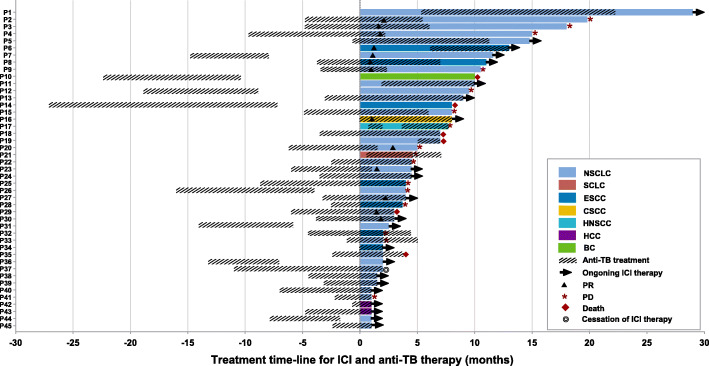
Treatment time-line for ICI and anti-tuberculosis in patients with tumor and active tuberculosis. TB tuberculosis, ICI immune checkpoint inhibitor, NSCLC non-small cell lung cancer, SCLC small cell lung cancer, ESCC esophageal squamous cell carcinoma, CSCC cutaneous squamous cell carcinoma, HNSCC head and neck squamous cell carcinoma, HCC hepatocellular carcinoma, BC breast cancer, PR partial response, PD progression disease

Specifically, when focused on the active tuberculosis group treated with concurrent anti-PD-(L)1 and anti-tuberculosis therapy. Ten patients with NSCLC, 2 patients with esophageal squamous cell carcinoma (ESCC), and 1 patient with cutaneous squamous cell carcinoma (CSCC) had partial responses. The DCR was 86.8% while 17.2% patients showed progressive disease. Twenty-three patients were still receiving anti-PD-(L)1 treatment while 16 developed progressive disease and 6 died. One patient discontinued immunotherapy due to grade 3 pneumonitis (Fig. 3).

### Outcomes of tuberculosis after anti-PD-(L)1 monotherapy or in combination with anti-tuberculosis therapy

We next analyzed the response to anti-tuberculosis treatment in patients with malignant tumor and active tuberculosis. Of the 45 patients, most of them (37/45, 82.2%) started anti-tuberculosis before anti-PD-(L)1 therapy with a median interval time of 3.93 months (Fig. 3). There were 80% (36/45) patients who showed an overlap treatment period between anti-PD-(L)1 and anti-tuberculosis therapies. The median duration of anti-tuberculosis was 10.0 (95%CI, 8.01–11.99) months with 18 (33.3%) patients still receiving anti-tuberculosis treatment (Fig. 3).

After the treatment of concurrent anti-PD-(L)1 and anti-tuberculosis therapy, patients in active tuberculosis group revealed 83.3% (20/24) sputum negative conversion, 2 remained positive, 2 retreated smear-positive, and 21 patients maintained negative sputum smear during the whole treatment. Meanwhile, 93.3% (42/45) patients showed radiographic tuberculosis absorption; however, 2 patients suffered from tuberculosis relapse after stopping anti-tuberculosis treatment (Additional file 1: Table S1 and Table 2).

**Table 2:** Characteristics and outcome of tuberculosis when treated with anti-PD(L)-1 or combined with anti-tuberculosis

	Characteristics and outcome of tuberculosis	Tumor and active TB (***n***, %)	Tumor and latent TB (***n***, %)	Tumor and obsolete TB (***n***, %)
**Before anti-PD(L)-1 or anti-TB therapy**	**IGRA**			
	Positive	29 (64.4)	21 (100)	0
	Negative	7 (15.6)	0	0
	Unknown	9 (20.0)	0	32 (100)
	**Sputum**			
	Positive	24 (53.3)	0	0
	Negative	21 (46.7)	21 (100)	32 (100)
	Unknown	0	0	0
	**Gene Xpert**			
	Positive	28 (62.2)	0	0
	Negative	11 (24.5)	21 (100)	0
	Unknown	6 (13.3)	0	32 (100)
	**Imaging features**			
	Secondary PTB	39 (86.7)	0	0
	Hematogenous PTB	1 (2.2)	0	0
	Tuberculous pleuritis	5 (11.1)	0	0
	Obsolete PTB	0	0	32 (100)
	Non-TB lesions	0	21 (100)	0
**After anti-PD(L)-1 or anti-TB therapy**	**Sputum conversion**			
	Sputum negative conversion	20 (44.4)	0	0
	Sputum positive conversion	2 (4.4)	0	1 (3.1)
	Persistent positive	2 (4.4)	0	0
	Persistent negative	21 (46.7)	21 (100)	0
	Unknown	0	0	31 (96.9)
	**Radiographic response**			
	TB absorption	42 (93.3)	NA	0
	TB progression	0	NA	1 (3.1)
	TB calcification	0	NA	31 (96.9)
	Unknown	3 (6.7)	NA	0
	**TB relapse or induction**			
	Yes	2 (4.4)	0	1 (3.1)
	No	43 (95.6)	21 (100)	31 (96.9)

Abbreviation: *IGRA* interferon gamma-released assay, *NA* not available, *PTB* pulmonary tuberculosis

For the latent tuberculosis group, according to the WHO consolidated guidelines on tuberculosis [25], none of these patients were in the population recommended for preventive treatment. All of these patients showed persistent bacteriologic and radiographic negative except for IGRA-positive during the treatment of anti-PD-(L)1.

For those with obsolete tuberculosis, 96.9% (31/32) patients showed stable radiographic tuberculosis calcification during the treatment of anti-PD-(L)1 while one patient eventually developed into secondary tuberculosis 1 year after anti-PD-(L)1 therapy (Table 2).

### Safety of concurrent anti-PD-(L)1 and anti-tuberculosis therapy

We furtherly analyzed the safety profile during the anti-PD-(L)1 and anti-tuberculosis treatment in the active tuberculosis group. The number of cases in treatment-related adverse events (TRAEs) is shown in Table 3. Thirty-three (73.3%) TRAEs (grade 1 [*n*=12], grade 2 [*n*=15], and grade 3–5 [*n*=6]) occurred following concurrent anti-PD-(L)1 and anti-tuberculosis treatment. The most common TRAEs were hematotoxicity (*n*=27), hepatic toxicity (*n*=4), rash (*n*=4), and fatigue (*n*=3). The grade 3–5 TRAEs included 5 hemototoxicity and 1 pneumonitis. Patient suffered grade 3 pneumonitis leading to the discontinuation of immunotherapy. Meanwhile, there was no unexpected toxic effects during the anti-PD-(L)1 and anti-tuberculosis treatment.

**Table 3:** Treatment-related adverse events during anti-PD(L)-1 and anti-tuberculosis treatment (*N*=45)

TRAEs	Any grade	Grade 1	Grade 2	Grade 3-5
**Any event**	33 (73.3)	12 (26.7)	15 (33.3)	6 (13.3)
**Hematotoxicity**	27 (60)	8 (17.8)	14 (31.1)	5 (11.1)
**Hepatic toxicity**	4 (8.9)	3 (6.7)	1 (2.2)	0
**Pneumonitis**	1 (2.2)	0	0	1 (2.2)
**Gastrointestinal toxicity**	2 (4.4)	2 (4.4)	0	0
**Rash**	4 (8.9)	3 (6.7)	1 (2.2)	0
**Fatigue**	3 (6.7)	2 (4.5)	1 (2.2)	0
**Endocrine toxicity**	1 (2.2)	1 (2.2)	0	0
**Cardiovascular toxicity**	0	0	0	0
**Any event leading to discontinuation of ICI**	1 (2.2)	0	0	1 (2.2)
**Any event leading to death**	0	0	0	0

Abbreviation: *TRAEs* Treatment-related adverse events, *ICI* immune checkpoint inhibitors

### Cases presentation

**Fig. 4:**
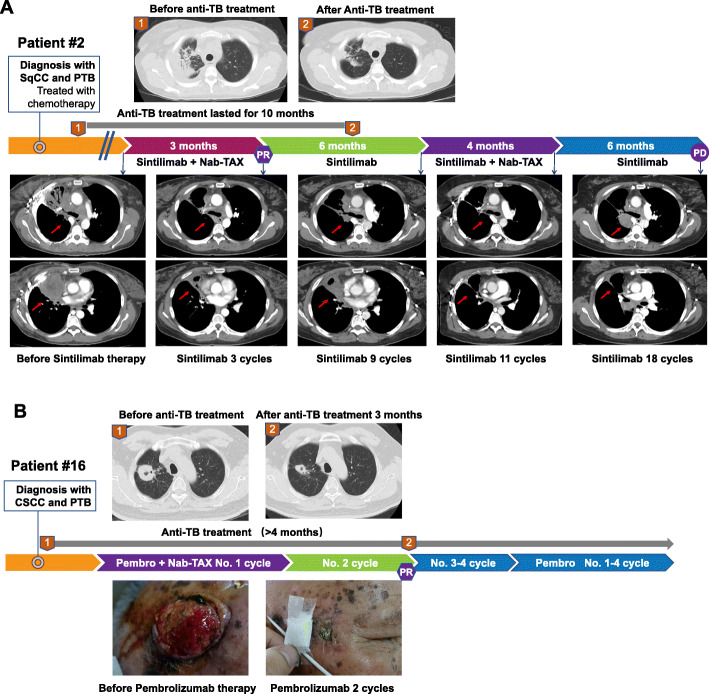
Representative cases and treatment response. **A** Patient #2 who was diagnosed with lung squamous cell cancer (SqCC) and pulmonary tuberculosis (PTB) received anti-tuberculosis treatment for 5 months followed by immunotherapy. **B** Patient #16 with cutaneous squamous cell carcinoma (CSCC) and PTB started anti-tuberculosis treatment for 1 month followed by immunotherapy

Herein, we presented 2 representative cases with concurrent anti-PD-(L)1 and anti-tuberculosis therapy in this cohort. Patient #2 who was diagnosed with lung squamous cell cancer and PTB received anti-tuberculosis treatment for 5 months followed by immunotherapy. After 10 months of anti-tuberculosis treatment, the patient exhibited significant radiographic response of tuberculosis foci and sputum conversion. After 3 cycles of second-line treatment of sintilimab plus chemotherapy, the patient achieved PR and developed progressive disease in the 18th cycle (Fig. 4A). Patient #16 with CSCC and PTB started anti-tuberculosis treatment for 1 month followed by immunotherapy. After 3 months of anti-tuberculosis treatment, the patient showed radiographic response and sputum conversion, and after 2 cycles of first-line treatment of pembrolizumab plus chemotherapy, the patient achieved PR and was still under ongoing immunotherapy (Fig 4B)

## Discussion

To our knowledge, this is the first real-world cohort study with the largest sample size to evaluate the efficacy and safety of anti-PD-(L)1 treatment in patients with coexisting malignancy and tuberculosis, as well as the efficacy of anti-tuberculosis treatment in those with active tuberculosis. Previous studies have reported individual cases, but there was no systematic cohort study [26–28]. This study suggests that these patients could benefit from anti-PD-(L)1 immunotherapy while achieving unimpaired anti-tuberculosis therapy. The results of this study provide reasonable clinical evidence for the treatment of patients with coexisting malignant tumor and tuberculosis, and the concurrent anti-PD-(L)1 and anti-tuberculosis therapy in patients with active tuberculosis.

Previous studies have identified specific immune activation and immune microenvironment alteration during tuberculosis infection [29, 30]; however, whether tuberculosis affected the tumor microenvironment and anti-tumor therapy was unknown. We furtherly analyzed the immune microenvironment in the resected tumor specimens from lung cancer patients with active or obsolete tuberculosis. The results revealed that patients with active tuberculosis had higher rate of high expression of PD-L1 and CD8+ lymphocyte infiltration compared to those with obsolete tuberculosis or single lung cancer (Additional file 2: Fig. S1). These results, to some extent, supported that patients with coexisting malignant tumors and active tuberculosis may be the potential candidates who will benefit from PD-1 blockade immunotherapy due to their inflammatory microenvironment.

The implication of PD-1/PD-L1 pathway has been confirmed in the pathophysiology of tuberculosis in preclinical studies [17–19, 31]. PD-1-deficient mice exhibited significant sensitivity to M.tuberculosis infection and survival reduction [19]. In addition, the infected PD-1-deficient mice developed severe necrotic pneumonia with marked elevation of serum proinflammatory cytokines [17]. These results show that the PD-1/PD-L1 pathway is involved in the occurrence and development of tuberculosis. Furthermore, both acute and reactivated tuberculosis have been described in patients undergoing treatment with anti-PD-1 in previous studies [28, 32, 33]. The view that tuberculosis could develop in cancer patients receiving immunotherapy has been represented in some studies [34]. However, the risk of reactivation of latent M tuberculosis or primary tuberculosis infection during ICI therapy is still unknown and clinicians are alerted to the development of active tuberculosis during immunotherapy. Thus, we aimed to address this clinical issue. In the latent tuberculosis group, none of these patients developed active tuberculosis after anti-PD-(L)1 therapy, which was in line with another retrospective study in which none of the anti-PD-1 regimen treated cancer patients with positive IGRA testing developed active tuberculosis [17]. In the active tuberculosis group, the combination of anti-PD-(L)1 and anti-tuberculosis treatment was safe but there were 2 patients with tuberculosis relapse; In the obsolete group, there was 1 patient with tuberculosis relapse. For these patients with tuberculosis relapse, it might be because of the reduced immunity in the condition of suffering from malignancy or the irregular anti-tuberculosis therapy due to the anti-PD-(L)1 treatment. Notably, 6 patients in our cohort initiated anti-tuberculosis therapy after anti-PD-(L)1 therapy (Fig 3). To explore whether they developed tuberculosis during anti-PD-(L)1 therapy, we reviewed their imaging data and found that suspicious tuberculosis lesions had already existed before anti-PD-(L)1 therapy and these lesions enlarged after anti-PD-(L)1 therapy but reduced after anti-tuberculosis treatment, resulting in a pseudo progression (Additional file 2: Fig. S2). These results indicated that the tuberculosis of these patients might be not caused or reactivated by anti-PD-(L)1 agents. Therefore, we found no evidence that immunotherapy induced tuberculosis occurrence or reactivation. Nevertheless, clinical data of large sample size is still necessary in the future for further verification.

Another important clinical question is when is the best timing to initiate anti-PD-(L)1 therapy in the context of active tuberculosis. ICI therapy was discontinued, temporarily interrupted, continued, or not specified when patients received anti-tuberculosis treatment in previous reported cases [17, 26, 28, 35, 36]. In our study, anti-tuberculosis therapy is mainly started about 4 months before immunotherapy. This treatment paradigm was similar to the previous anti-tuberculosis and anti-tumor therapies, so as to achieve sputum conversion via anti-tuberculosis treatment for 2 to 3 months before anti-tumor treatment. However, to date, there is still no relevant guideline to clarify the order and interval between anti-tuberculosis and anti-tumor treatments.

This study has several limitations. First, the relatively short time for follow-up precludes meaningful survival analysis, and further toxicities may emerge over time. Second, it is a retrospective study. Thus, further prospective clinical studies are required to clarify the indications and management of immunotherapy for patients with coexisting tuberculosis and malignancy.

## Conclusions

This study demonstrated that patients with coexisting malignant tumor and tuberculosis showed relatively higher overall response and benefit equally among three groups from anti-PD-(L)1 therapy. Furthermore, anti-tuberculosis treatment was well-controlled for those with active tuberculosis. Notably, the combination of anti-PD-(L)1 and anti-tuberculosis therapy was well-tolerated without unexpected toxic effects. Our results provide the clinical evidence for the application of PD-(L)1 inhibitors in cancer patients with tuberculosis, rendering more available treatment options for these patients. Clinicians may judiciously consider the anti-PD-(L)1 treatment in patients with malignancy and tuberculosis.

## Supplementary Information

**Additional file 1: Table S1.** Efficacy of anti-TB treatment when combined with anti-PD(L)-1 therapy.

**Additional file 2: Figures S1-S2. Fig S1.** The correlation between tuberculosis status and tumor immune microenvironment. **Fig S2.** Representative cases with suspicious tuberculosis before anti-PD-1 immunotherapy.

## Data Availability

Data relevant to the study are included in the article or uploaded as supplementary information. Any additional data pertaining to this manuscript are available from the corresponding author upon reasonable request.

## Abbreviations

NSCLC: Non-small cell lung cancer
SCLC: Small cell lung cancer
ESCC: Esophageal squamous cell carcinoma
CSCC: Cutaneous squamous cell carcinoma
HNSCC: Head and neck squamous cell carcinoma
HCC: Hepatocellular carcinoma
BC: Breast cancer
RC: Rectal cancer
ICI: Immune checkpoint inhibitors
IGRA: Interferon gamma-released assay
TB: Tuberculosis
PTB: Pulmonary tuberculosis
PFS: Progression-free survival
ORR: Objective response rate
TRAEs: Treatment-related adverse events
PD-1: Programmed cell death 1
PD-L1: Programmed cell death ligand 1
DOR: Duration of response
DCR: Disease control rate
