# Supplementary material for: Assessment of anti-PD-(L)1 for patients with coexisting malignant tumor and tuberculosis classified by active, latent, and obsolete stage
Source: BMC Med. 2021 Dec 20;19:322. doi: 10.1186/s12916-021-02194-z (PMC8686368; doi:10.1186/s12916-021-02194-z)
Supplement: Supplementary file 1 — Additional file 1: Table S1. Efficacy of anti-TB treatment when combined with anti-PD(L)-1 therapy. [file 12916_2021_2194_MOESM1_ESM.docx]

| **Table S1.** Efficacy of anti-TB treatment when combined with anti-PD(L)-1 therapy | | | | |
| --- | --- | --- | --- | --- |
| **Patients** | **Duration of anti-TB (month)** | **Sputum negative conversion** | **Radiographic response** | **TB relapsed** |
| **P01** | 17 | Yes | Yes | No |
| **P02** | 10 | Yes | Yes | No |
| **P03** | 11 | - | Yes | No |
| **P04** | 12 | - | Yes | No |
| **P05** | 12 | - | Yes | No |
| **P06** | 7+ | No | Yes | Yes |
| **P07** | 7 | - | Yes | No |
| **P08** | 11 | - | Yes | No |
| **P09** | 6 | Yes | Yes | No |
| **P10** | 12 | Yes | Yes | No |
| **P11** | 8 | - | Yes | No |
| **P12** | 10 | Yes | Yes | No |
| **P13** | 12+ | - | Yes | No |
| **P14** | 20 | - | Yes | No |
| **P15** | 11 | - | Yes | No |
| **P16** | 9+ | Yes | Yes | No |
| **P17** | 4+ | Yes | Yes | No |
| **P18** | 10 | Yes | Yes | No |
| **P19** | 2 | No | Yes | No |
| **P20** | 8 | Yes | Yes | No |
| **P21** | 7+ | Yes | Yes | No |
| **P22** | 7 | - | Yes | No |
| **P23** | 6 | - | Yes | No |
| **P24** | 8+ | - | Yes | No |
| **P25** | 13+ | Yes | Yes | No |
| **P26** | 12 | - | Yes | No |
| **P27** | 7+ | - | Yes | No |
| **P28** | 6 | Yes | Yes | No |
| **P29** | 9 | No | Yes | Yes |
| **P30** | 7.5+ | No | Yes | No |
| **P31** | 8 | - | Yes | No |
| **P32** | 9+ | Yes | Yes | No |
| **P33** | 6 | Yes | Yes | No |
| **P34** | 2+ | Yes | NA | No |
| **P35** | 6 | - | NA | No |
| **P36** | 6 | Yes | Yes | No |
| **P37** | 13+ | - | Yes | No |
| **P38** | 6 | Yes | Yes | No |
| **P39** | 5+ | - | Yes | No |
| **P40** | 8+ | Yes | Yes | No |
| **P41** | 3+ | - | Yes | No |
| **P42** | 1.5+ | Yes | NA | No |
| **P43** | 5.5+ | Yes | Yes | No |
| **P44** | 7 | - | Yes | No |
| **P45** | 3+ | - | Yes | No |
| Abbreviation: +, ongoing anti-TB treatment; NA, not available; -, Persistent negative. | | | | |
