# Supplementary figures and images for: Assessment of anti-PD-(L)1 for patients with coexisting malignant tumor and tuberculosis classified by active, latent, and obsolete stage
Source: BMC Med. 2021 Dec 20;19:322. doi: 10.1186/s12916-021-02194-z (PMC8686368; doi:10.1186/s12916-021-02194-z)

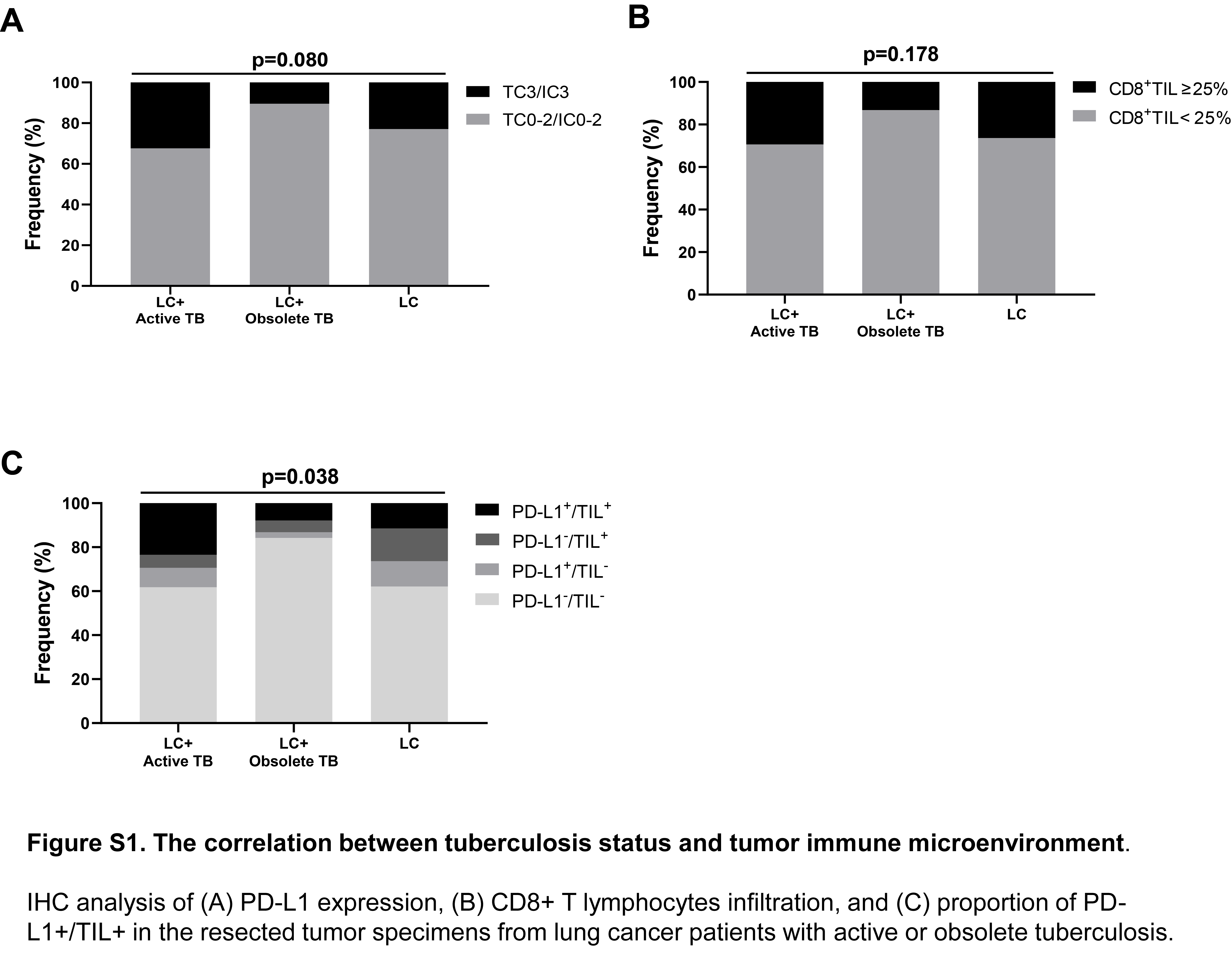

Supplement: Supplementary file 2 — Additional file 2: Figures S1-S2. Fig S1. The correlation between tuberculosis status and tumor immune microenvironment. Fig S2. Representative cases with suspicious tuberculosis before anti-PD-1 immunotherapy. [file 12916_2021_2194_MOESM2_ESM.zip › Additional file 2/Figure S1R1.tif]

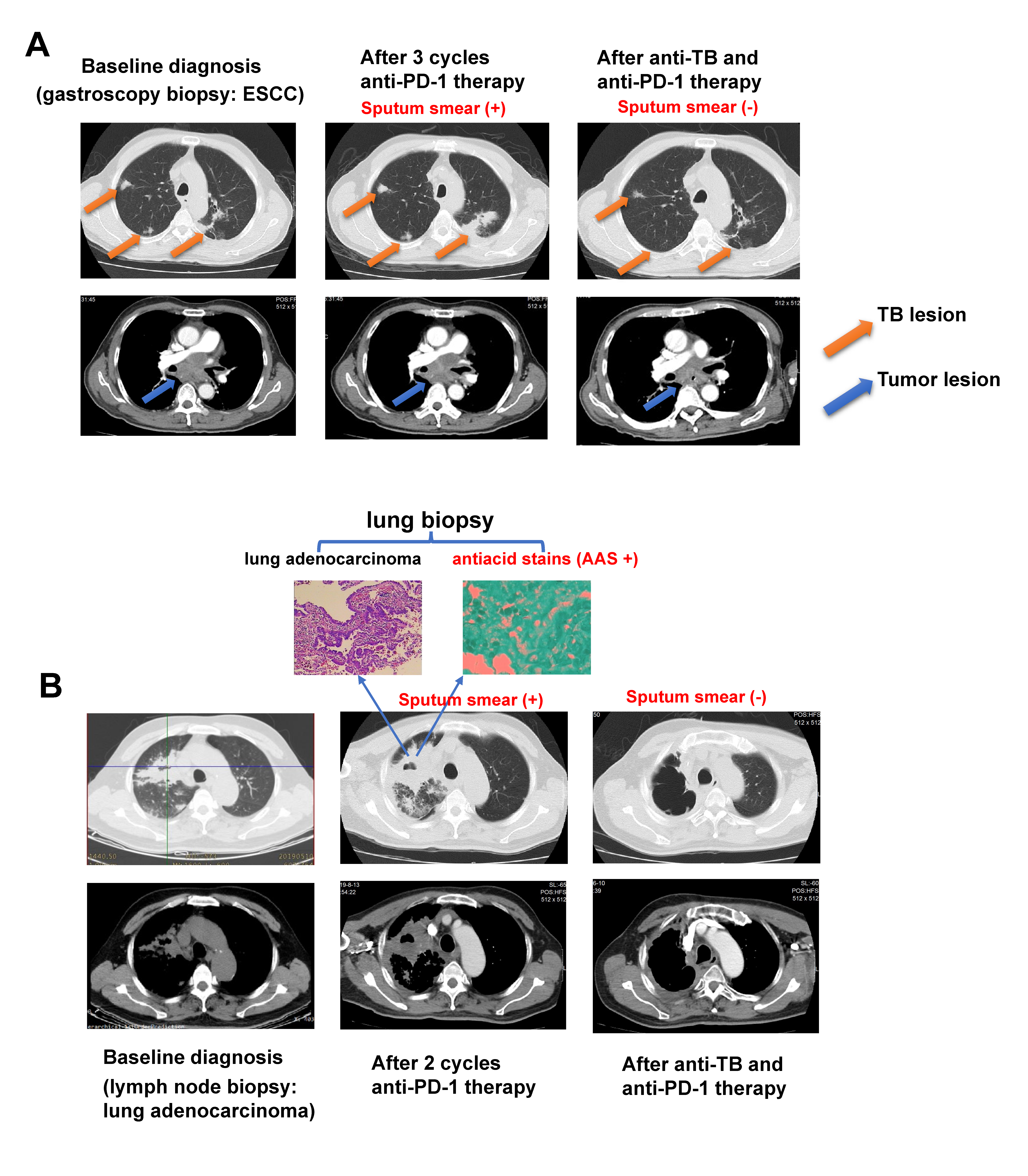

Supplement: Supplementary file 2 — Additional file 2: Figures S1-S2. Fig S1. The correlation between tuberculosis status and tumor immune microenvironment. Fig S2. Representative cases with suspicious tuberculosis before anti-PD-1 immunotherapy. [file 12916_2021_2194_MOESM2_ESM.zip › Additional file 2/Figure S2R1.tiff]
